# Sensitizing human multiple myeloma cells to the proteasome inhibitor bortezomib by novel curcumin analogs

**DOI:** 10.3892/ijmm.2011.814

**Published:** 2011-10-13

**Authors:** TASKEEN MUJTABA, JYOTI KANWAR, SHENG BIAO WAN, TAK HANG CHAN, Q. PING DOU

**Affiliations:** 1 The Developmental Therapeutics Program, Barbara Ann Karmanos Cancer Institute, and Departments of Oncology, Pharmacology and Pathology, School of Medicine, Wayne State University, Detroit, MI, USA; 2 Department of Applied Biology and Chemistry, The Polytechnic University of Hong Kong, Hung Hum, Hong Kong SAR, P.R. China; 3 Department of Chemistry, McGill University, Montreal, Quebec H3A 2K6, Canada

**Keywords:** multiple myeloma, bortezomib, curcumin, curcumin analogs, drug resistance

## Abstract

The proteasome plays a vital role in the degradation of proteins involved in several pathways including the cell cycle, cellular proliferation and apoptosis and is a validated target in cancer treatment. Bortezomib (Velcade^®^, PS-341) is the first US FDA approved proteasome inhibitor anticancer drug used in the treatment of refractory multiple myeloma. In spite of its improved efficacy compared to alternative therapies, about 60% of patients do not respond to bortezomib due to the emergence of resistance. We hypothesized that novel small molecules could enhance the proteasome-inhibitory and anticancer activities of bortezomib in resistant multiple myeloma cells *in vitro* and *in vivo*. The dietary polyphenol curcumin has been shown to exert anti-cancer activity in several cancer cell lines, but the effects of curcumin in solid tumors have been modest primarily due to poor water solubility and poor bioavailability in tissues remote from the gastrointestinal tract. Here we show that the water-soluble analog of curcumin #12, but not curcumin, in combination with bortezomib could enhance the proteasome-inhibitory effect in multiple myeloma cells. Furthermore, the sensitivity of the myeloma cells to cytotoxic killing in the presence of otherwise sublethal concentrations of bortezomib was enhanced by incubation with the curcumin analog #12. These findings justify further investigation into those combinations that may yield potential therapeutic benefit.

## Introduction

Multiple myeloma (MM) is a neoplasm of mature clonal B cells and is the second most common hematological malignancy after non-Hodgkin’s lymphoma. It accounts for approximately 15% of all lymphohematopoietic cancers. Incidence of MM increases with age and its rate of occurrence is higher in men. Despite the availability of new drugs MM still remains an incurable disease. Current cancer therapies are intended to selectively target specific biomarkers that are altered in the malignant phenotype to dampen tumor progression, which is limited by many factors. Alternatively, targeting the proteasome, which regulates multiple cellular events, represents one of the most successful anticancer strategies.

Bortezomib (Velcade^®^, PS-341) is the first US food and drug administration (FDA) approved proteasome inhibitor. It has shown remarkable success in the treatment of hematological malignancies, however about 60% of patients do not respond to bortezomib due to the emergence of resistance. Some of the molecular mechanisms for resistance have been found to be the overexpression of the β5 subunit of the proteasome (1) overexpression of the anti-apoptosis protein Bcl-2 (2), and high secretion of GRP-78, a chaperone protein of the unfolded protein response (3). In B-cell lymphomas overexpression of heat shock protein (HSP)27, HSP70, HSP90 and T-cell factor 4 is associated with bortezomib resistance (4). In solid tumor cell lines (lung, cervical and colon) bortezomib treatment had minimal effects as it induced the formation of stress granules (5). Proteasome inhibitor-resistant constitutive activation of NF-κB has also been reported (6). Targeting these resistance mechanisms could therefore increase the efficacy of proteasome inhibition by bortezomib. Another potential limitation of bortezomib therapy is its interaction with several natural products including polyphenols containing vicinal diols that block its activity (7). Overcoming these challenges requires identification of mechanisms that confer sensitivity to proteasome inhibition and identification of a novel class of agents, which can be safely used in combination with bortezomib.

A potential prospect is to use natural compounds that do not interact with bortezomib to sensitize tumors to bortezomib therapy. Several naturally occurring products have great potential as chemopreventive agents. Curcumin is one such compound that has attracted much interest due to its proven pharmacological safety and its many favorable biological activities such as anti-inflammation, anti-oxidation, chemopreventive and chemotherapeutic activities (8). Recently, the tumor cellular proteasome has been reported as an important target of curcumin (9).

The anticancer effects of curcumin, both alone and in combination with chemotherapeutics are widely supported by several studies (10–12). Curcumin in combination with bortezomib has been reported to synergistically induce apoptosis in human multiple myeloma U266 cells (13). Curcumin was reported to potentiate the effect of bortezomib against human multiple myeloma in a nude mice model (14). These observations therefore suggest that a superior therapeutic index may be achieved with curcumin when used in combination and could be advantageous in the treatment of refractory tumors.

**Figure 1:**
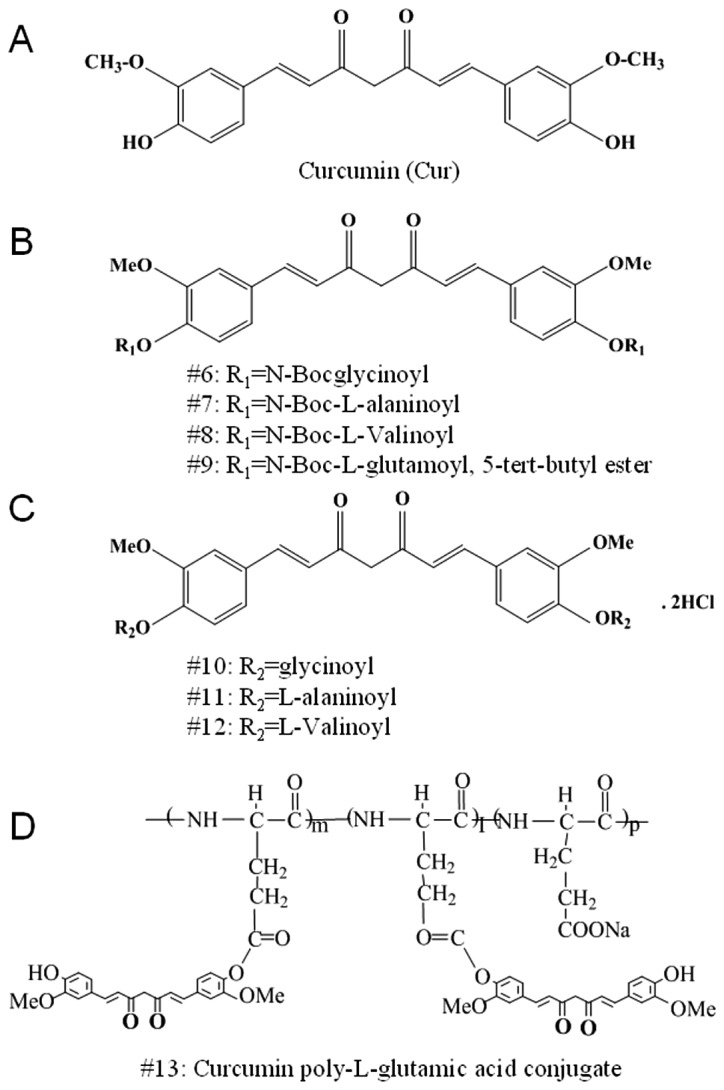
Structures of (A) curcumin and (B–D) amino acid-conjugated curcumin analogs 6–13.

Unfortunately, the *in vivo* biological activities of curcumin were found to be greatly reduced due to its low solubility and bioavailability in tissues remote from the gastrointestinal tract (10–12). In an effort to enhance the bioavailability of curcumin, we synthesized several novel analogs of curcumin, and we recently reported that water soluble amino acid conjugates of curcumin are potent proteasome inhibitors and showed a potent antiproliferative effect in several human cancer cell lines (15). Docking studies of these conjugates suggested that they may serve as the water soluble analogs of curcumin (15). In the present study, we examined the ability of the curcumin analogs 6–13 (Fig. 1) (15) to sensitize multiple myeloma cells to otherwise sublethal concentrations of bortezomib and enhance its proteasome-inhibitory anticancer activities.

## Materials and methods

### Cell culture

Human multiple myeloma Arp cell line, kindly provided by Dr Ramesh Batchu (Wayne State University), were grown in RPMI-1640 medium supplemented with 10% FBS, 100 U/ml of penicillin, and 100 μg/ml of streptomycin. Cells were maintained at 37°C in 5% CO_2_.

### Chemicals

Amino acid conjugates 6–13 of curcumin were prepared as previously reported (15).

### Inhibition of purified 20S and cellular 26S proteasome activity by curcumin and its analogs

Arp multiple myeloma cells were treated with curcumin or amino acid conjugated water soluble curcumin analogs either alone or in combination with bortezomib for 48 h at 37°C. Cell extracts from these cells were then used to measure the proteasomal chymotryptic (CT) activity, trypsin like activity, PGPH activity and caspase-3 activity. All activities in both the purified proteasome and the cellular proteasome were measured following a previously described protocol (16).

### Cell proliferation and viability

Myeloma cells were grown in 96-well plates. Cells were treated with the indicated concentrations of curcumin or the curcumin analogs alone or in combination with bortezomib for 24–48 h followed by an MTT assay to measure cell proliferation and cell viability (17).

### Immunoblotting

Multiple myeloma cells were treated with either curcumin or its analogs both alone and in combination with bortezomib at 5 nM concentration for 48 h. Cells were then harvested for the preparation of lysates. Equal amounts of protein (40 μg/lane) were electrophoresed on SDS-PAGE gels, transferred to nitrocellulose, and probed with the indicated antibodies as previously described (17).

## Results

### Curcumin analogs enhance the inhibitory effect of bortezomib on the purified 20S proteasome

**Figure 2:**
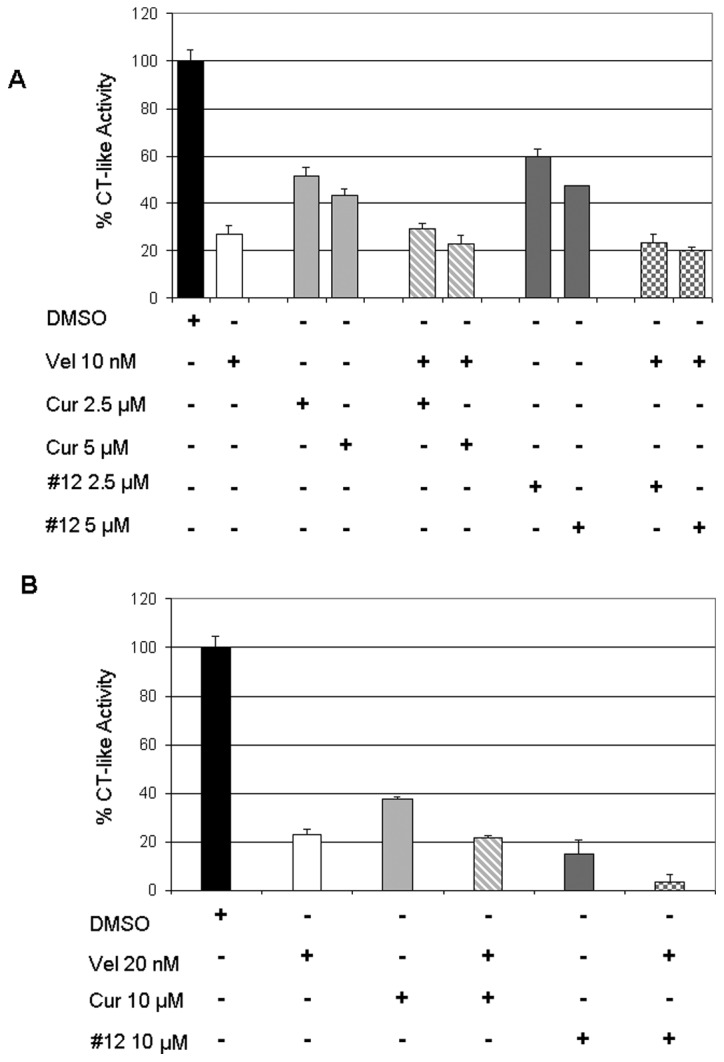
Curcumin analogs inhibit CT activity of the purified 20S proteasome. (A) Curcumin analog #12 was examined at 2.5 and 5 μM both alone and in combination with bortezomib (Vel) at 10 nM. DMSO and curcumin were used as controls. (B) The curcumin analog #12 was examined at 10 μM both alone and in combination with bortezomib (Vel) at 20 nM. Error bars indicate SD of the mean for all treatments in triplicate.

To determine if the curcumin analogs could add to or inhibit bortezomib’s activity, the analogs alone or in combination with bortezomib were evaluated for their ability to inhibit the purified 20S proteasome CT activity. Curcumin analogs were examined at 2.5, 5 and 10 μM both alone and in combination with bortezomib (Vel) at 10 nM. Curcumin analogs 6, 7, 8 and 9 did not significantly inhibit CT-activity of purified 20S proteasome when compared with bortezomib. They did not add to or inhibit bortezomib activity in combination studies (data not shown). On the other hand, the water soluble analogs of curcumin 10, 11, 12 and 13 alone potently inhibited proteasomal CT activity (data not shown). When used in combination with 10 nM bortezomib the effect was additive and they further lowered the CT activity compared with bortezomib alone. The most pronounced effect was observed with analog #12 (Fig. 2). In particular, #12 at 10 μM in combination with bortezomib at 20 nM was appreciably more effective in suppressing CT-like activity than bortezomib at 20 nM alone. In comparison, curcumin at 10 μM in combination with bortezomib at 20 nM did not have a pronounced inhibitory effect under our experimental conditions.

### Curcumin analogs enhance the proteasome-inhibitory effect of bortezomib in human myeloma cells

**Figure 3:**
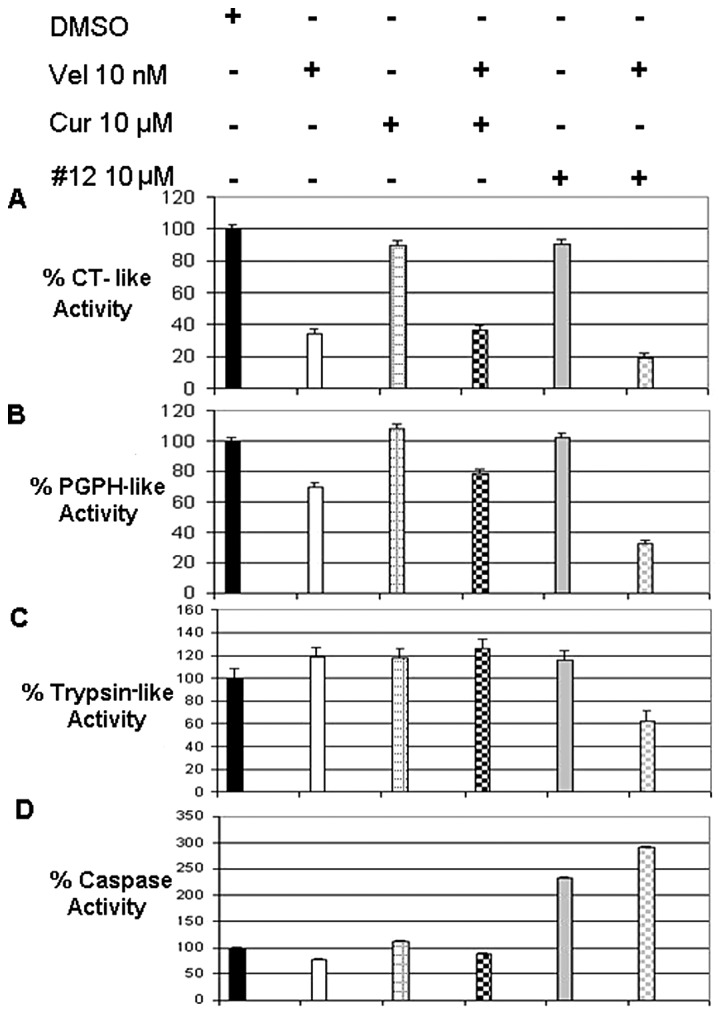
Effect of curcumin analogs plus bortezomib on various proteasome activities and caspase-3 activity. Protein extracts from Arp myeloma cells after treatment with analog #12 and bortezomib at the indicated concentrations for 48 h were examined for all three proteasome activities and caspase-3 activity. Analog #12 when combined with 10 nM bortezomib significantly inhibited chymotryptic (CT)-, PGPH- and trypsin-like activities of the proteasome and also increased the caspase-3 activity in these cells. Error bars indicate SD of the mean of all treatment values in triplicate.

We next determined if the curcumin analogs could add to bortezomib’s activity in intact myeloma cells. Arp cells were co-treated with curcumin analogs 10, 11, 12 and 13 at 10 μM for 48 h both alone and in combination with bortezomib at 10 nM, and cell extracts were then prepared and assayed for three proteasomal activities (CT-, PGPH- and trypsin-like). None of the analogs alone at 10 μM could inhibit the activities of the proteasome in these cells (data not shown). Analog #12, but not curcumin, enhanced the effects of bortezomib and the co-treatment showed potent inhibitory effects on CT-, PGPH- and trypsin-like activities of the proteasome (Fig. 3A–C).

### Curcumin analogs enhance bortezomib-mediated proliferation inhibition and apoptosis induction in multiple myeloma cells

**Figure 4:**
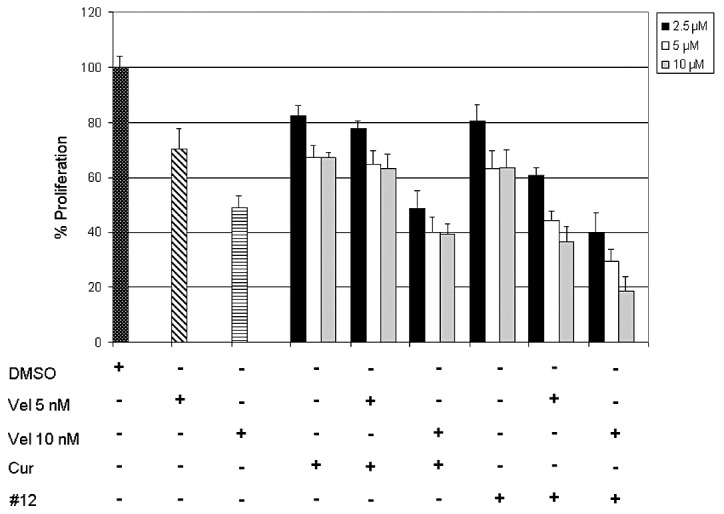
Curcumin analogs inhibit cell proliferation. Arp multiple myeloma cells were treated with curcumin analog #12 at the indicated concentrations both alone and in combination with bortezomib (Vel) at 5 and 10 nM. Analog #12 showed potent inhibition of cell proliferation both alone and in combination with bortezomib (Vel).

We then determined whether the observed enhancement of the proteasome-inhibitory effect of bortezomib by the water soluble curcumin analog #12 was associated with an increase in the growth inhibition and apoptosis induction in these cells. Water soluble curcumin analogs were tested for inhibition of cell proliferation in ARP multiple myeloma cells by the MTT assay at various concentrations, (2.5, 5, and 10 μM) both alone and in combination with bortezomib at 5 and 10 nM. DMSO and curcumin were used as controls. Curcumin analogs 10, 11, 13 and especially 12 showed inhibition of cell proliferation but further inhibition was observed when they were combined with bortezomib at 5 and 10 nM (Fig. 4 and data not shown). In the cells co-treated with analog #12 and bortezomib (Fig. 3), caspase-3 activity was also enhanced (Fig. 3D). The combination of curcumin and bortezomib had no such enhancing effect. These results show that the sensitivity of Arp myeloma cells to cytotoxic killing in the presence of otherwise sublethal concentrations of bortezomib was enhanced by incubation with curcumin analog #12.

### Curcumin analog #12 increases cellular proteasome-inhibitory and apoptosis-inducing activity of bortezomib in a time-dependent manner

**Figure 5:**
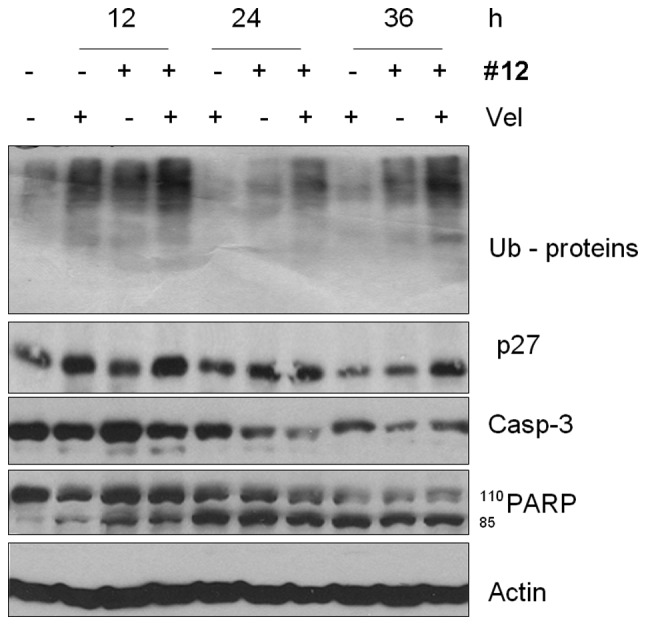
Immunoblot experiment with protein extracts from Arp multiple myeloma cells after treatment with analog #12 and bortezomib at the indicated concentrations for various time points. Increased accumulation of proteasome target protein p27 and ubiquitinylated (Ub) proteins and increased PARP and caspase 3 (Casp-3) cleavages at early time points was observed in combination treatments compared to bortezomib (Vel) alone.

To understand the mechanism of sensitization by the curcumin analog #12, we performed a kinetic experiment using a combination of 10 μM of analog #12 and 5 nM bortezomib in Arp cells. Western blot analysis of the co-treatment revealed a time-dependent increase in the accumulation of ubiquitinylated proteins and the proteasome target protein p27 in co-treated cells compared with each treatment alone (Fig. 5). Compared with bortezomib treatment alone, co-treatment with analog #12 also increased PARP and caspase 3 cleavages at early time points (Fig. 5). These results suggest that analog #12 induced apoptosis in the presence of otherwise sublethal concentrations of bortezomib.

## Discussion

Cancer treatment requires simultaneous suppression of multiple signaling/survival pathways. Therefore, emphasis has been laid on combinational therapies that may be more effective than single agents. Previously, curcumin has been reported to synergize with bortezomib and potentiate the apoptotic effects in human multiple myeloma U266 cells via down regulating NF-κB and its regulated gene products (13). We have previously reported that curcumin can potentially inhibit the proteasome activity which is required for the subsequent activation of the NF-κB pathway (9). Although curcumin is very effective, it is poorly water soluble and less bioavailable in tissues remote from the gastrointestinal tract. In our efforts to enhance its bioavailability we synthesized amino acid-conjugated curcumin analogs and have previously reported them to be potent inhibitors of the proteasome and cell proliferation (15). Based on our previous work with curcumin analogs we further investigated the efficacy of these analogs as chemosensitizers, in combination with bortezomib.

Consistent with our earlier observations in human colon cancer and prostate cancer cells, water soluble amino acid-conjugated curcumin analogs showed potent inhibition of the proteasome and cell proliferation in Arp multiple myeloma cells. Among the curcumin analogs, combination of analog #12 and bortezomib had a more additive effect on proteasome inhibition, proliferation inhibition, and apoptosis induction in these cells. To confirm that the additive effects of this analog were possibly through inhibition of the cellular proteasome activity in these cells we investigated the various proteasomal activities. Our results from the proteasomal activity assays indicate that analog #12 of curcumin when combined with bortezomib could extensively inhibit the CT-like, trypsin-like and PGPH-like activities of the cellular proteasome (Fig. 3). Furthermore, the combination of analog #12 with bortezomib results in a considerable increase in caspase activity. An increase in caspase-3 cleavage at earlier time points in the combination treatments could trigger apoptosis in these cells (Fig. 5).

In chemoresistant multiple myeloma cells from patients, the NF-κB pathway has been shown to be constitutively active compared with chemosensitive lines and has been associated with resistance to anticancer therapy (6). However, the activation of NF-κB has been shown to be prevented by proteasome inhibitors (18). Thus, in the ARP human MM cell line, our results suggest that analog #12 of curcumin could induce apoptosis in the presence of otherwise sublethal concentrations of bortezomib possibly through the down-regulation of the NF-κB pathway as a downstream effect of proteasome inhibition, which will be investigated in the near future. Therefore, it is possible that water soluble curcumin analog #12 with increased bioavailability may be useful as potential replacement of curcumin and could be used in combination to bortezomib. Validation of such possibility using animal models is warranted.
